# Parameters Optimization of Catalytic Tubular Nanomembrane-Based Oxygen Microbubble Generator

**DOI:** 10.3390/mi11070643

**Published:** 2020-06-29

**Authors:** Sumayyah Naeem, Farah Naeem, Jing Zhang, Jawayria Mujtaba, Kailiang Xu, Gaoshan Huang, Alexander A. Solovev, Yongfeng Mei

**Affiliations:** 1 State Key Laboratory for Modification of Chemical Fibers and Polymer Material Science and Engineering, Donghua University, Shanghai 201620, China; sumayyahnaeem@gmail.com (S.N.); farah11naeem2013@gmail.com (F.N.); 2 Department of Materials Science, Fudan University, Shanghai 200433, China; jawayria.m@icloud.com (J.M.); gshuang@fudan.edu.cn (G.H.); yfm@fudan.edu.cn (Y.M.); 3 College of Science, Donghua University, Shanghai 201620, China; 4 Department of Electronic and Engineering, Fudan University, Shanghai 200433, China; xukl@fudan.edu.cn

**Keywords:** oxygen, hydrogen peroxide, microreactor, catalytic, nanomembrane, microtube, bubble

## Abstract

A controllable generation of oxygen gas during the decomposition of hydrogen peroxide by the microreactors made of tubular catalytic nanomembranes has recently attracted considerable attention. Catalytic microtubes play simultaneous roles of the oxygen bubble producing microreactors and oxygen bubble-driven micropumps. An autonomous pumping of peroxide fuel takes place through the microtubes by the recoiling microbubbles. Due to optimal reaction–diffusion processes, gas supersaturation, leading to favorable bubble nucleation conditions, strain-engineered catalytic microtubes with longer length produce oxygen microbubbles at concentrations of hydrogen peroxide in approximately ×1000 lower in comparison to shorter tubes. Dynamic regimes of tubular nanomembrane-based oxygen microbubble generators reveal that this depends on microtubes’ aspect ratio, hydrogen peroxide fuel concentration and fuel compositions. Different dynamic regimes exist, which produce specific bubble frequencies, bubble size and various amounts of oxygen. In this study, the rolled-up Ti/Cr/Pd microtubes integrated on silicon substrate are used to study oxygen evolution in different concentrations of hydrogen peroxide and surfactants. Addition of Sodium dodecyl sulfate (SDS) surfactants leads to a decrease of bubble diameter and an increase of frequencies of bubble recoil. Moreover, an increase of temperature (from 10 to 35 °C) leads to higher frequencies of oxygen bubbles and larger total volumes of produced oxygen.

## 1. Introduction

Oxygen gas has broad applications in clean energy, medicine, chemistry, biology. However, conventional methods such as pressurized oxygen tanks, oxygen concentrators (i.e., separation from the air) and obtaining gas during water splitting suffer from high complexity and costs [1]. Catalytic nanomaterials that do not require wires and external energy sources to generate oxygen from sustainable reactions like decomposition of hydrogen peroxide are highly desirable and in high demand for multiple applications. The dynamic motion of catalytic nano/-microparticle-based motors have been widely investigated for their potential applications including environmental remediation [2,3,4,5], water cleaning [6,7], bio-sensing in motion [8,9,10], minimally invasive surgery, delivery of drugs [11,12,13,14,15], multiplexed immunoassays [16] and radioactive uranium preconcentration [17], to name a few examples. Several motive mechanisms have been discovered for micromotors such as self-electrophoresis, self-diffusiophoresis, dynamic surface tension and bubble recoil [3]. Particular attention attracted catalytic microtubes made of strain-engineered rolled-up inorganic nanomembranes due to efficient tubular geometry to confine reaction-diffusion processes and therefore, control the threshold hydrogen peroxide concentrations required for bubbles’ nucleation/generation [18]. For instance, it was demonstrated that 1 mm long Ti/Cr/Pt tubes require H_2_O_2_ concentration in approximately ×1000 times lower to generate O_2_ bubbles than 40 µm long tubes [19]. Subsequently, different catalysts (e.g., Ag) [20], fabrication methods (e.g., template electrosynthesis) [21] and tubular wall materials (e.g., graphene/MnO_2_ [22]) have been investigated. It is worthy to note that microtubes can produce bubbles from a single tubular opening (unidirectional regime) or both tubular openings (“overloaded”, bidirectional regime) [23]. Novel microfluidic techniques based on glass capillaries have been adopted to produce nanoparticle-shelled bubbles with catalytic shells [24]. There is a number of forces acting on recoiling microbubbles [25] as well as surfactants, which can reduce the surface tension of peroxide fuel and stabilize microbubbles [26,27]. Temperature increase leads to higher catalytic reaction rates. Previously it was reported that catalytic microtubes at 37 °C generate a significantly larger number of oxygen microbubbles [28]. Only until recently, it was realized that catalytic micromotors are interesting, not due to their motion, but also because of their efficient generation of potentially useful byproducts: water and oxygen during the decomposition of hydrogen peroxide in the presence of a catalyst. It was reported that higher concentrations of hydrogen peroxide and longer tubular lengths lead to higher frequencies of oxygen bubble recoil, however, a smaller total amount of oxygen is produced [29]. It was observed that the total gas generation rate is higher for tubes, which grow larger bubbles from the tubular opening, rather than generating bubbles inside of tubes. Since then, the template-assisted method has been adopted and the influence of salts on bubbles formation was investigated [30]. Heterogeneous micro-cavities or surface defects play an important role in energy reduction for nucleation and generation of bubbles as a result of gas supersaturation at the solid–liquid interface [31]. Besides catalytic particle size, catalytic surface curvature, i.e., convex, flat, concave, has a direct influence on the energy of bubble nucleation and growth [32]. Subsequently, proper design and understanding of a gas-producing micro-reactor during reaction-diffusion processes are required for the efficient production of oxygen.

Conventional oxygen generators suffer from high price, complex architectures, generation of undesirable byproducts or they depend on external energy supply. A development of facile preparation methods, containing earth-abundant catalysts, and producing oxygen at neutral pH, room temperature, low overpotentials during water splitting have received considerable attention [33,34,35]. An application of aqueous hydrogen peroxide to generate oxygen is a simple, cost-effective, and reliable method [36]. Mechanisms involving intermediate reaction products and kinetics using popular catalysts such as manganese oxide were previously explored [37]. An efficient parchment paper material for local wound oxygenation was demonstrated [38]. In part, the research direction is also driven towards the investigation of single compartment hydrogen peroxide clean fuel cells, such as with poly(3,4-ethylenedioxythiophene) cathodes [39], integration of micromotors to power gaseous hydrogen/oxygen fuel cells [40] and related chemo-mechano-electrical energy converters [41].

Herein, oxygen evolution is studied using Ti/Cr/Pd catalytic microtubes by varying microtube length, SDS surfactant, solution surface tension, and concentration of H_2_O_2_ as parameters influencing frequencies of oxygen bubbles and total amounts of produced gas. Larger concentrations of SDS surfactants lead to smaller oxygen microbubbles and less total volume of generated oxygen. For 45 µm long tubes, temperature is tested in the range 10 to 36 °C with higher temperature leading to increased rates of bubble recoil and total oxygen gas. This study offers a better understanding of how different parameters of catalytic tubular microreactors and composition of hydrogen peroxide chemical fuel influence oxygen bubbles frequencies, radius and total volume, which pave the way towards a practical portable oxygen generator.

## 2. Materials and Methods

### 2.1. Fabrication of Catalytic Microtubes

Previously reported fabrication process of rolled-up catalytic microtubes were used to synthesize the Ti/Cr/Pd tubes [23,29,42]. A photoresist ARP-3510 was deposited on 1-inch square Si (100) wafer by the spin coating method at 3500 rmp for 35 s and soft baked at 90 °C for 2 min. Patterns with dimensions 15–100 µm were exposed by UV-light with Karl Suss MA 56 Mask Aligner for 7 s. The developer 1:1 AR300-35 H_2_O solution was used to remove the light-exposed photoresist parts. The active layers of materials were deposited under the high vacuum. To obtain the on-chip rolled-up catalytic microtubes, 10/10/5 nm of each metal were deposited by tilted deposition at 60° angle. This method helps to obtain microtubes on the surface (on-chip) in a precise position. Pre-stressed multilayers self-roll-up into the assembled microtubes by dissolving the photoresist layer with 100% selectivity. To avoid the collapsing of rolled-up microtubes, a supercritical point dryer was used. To observe the catalytic behavior, microtubes were immersed into a surfactant aqueous solution, such as sodium dodecyl sulfate (Sigma-Aldrich, St. Louis, MI, USA), which reduces surface tension and stabilizes microbubbles during catalytic decomposition of hydrogen peroxide.

### 2.2. Measurements of Oxygen Microbubbles

Experiments of oxygen-generating microtubes were performed in the Petri dish containing different concentrations of surfactant solutions (in the range 2–10%) in distilled water. A silicon wafer with integrated tubes was immersed into the solution with added hydrogen peroxide (in the range 0.125–20%). Optical microscopy images were captured by the Olympus optical microscope and videos were recorded using a high-speed camera at 190 frames/s. Surface tension measurements of different concentrations of SDS surfactant solutions were carried out using the maximum bubble pressure method (tensiometer BP50). The cooling of the solution was controlled by the cooling pad and temperature was measured by the thermometer. The heating of the solution was controlled by the 5 V USB electric heating pad with a temperature-controlled system. Oxygen concentrations are measured in three different conditions, in the ambient air, a small size box with dimensions l × h × w= 8 × 6 × 6 cm and a large size box with dimensions l × h × w= 16 ×1 0 × 10 cm containing the bubble oxygen generator. Oxygen meter, temperature meter, and solution with the sample were inserted in the boxes. Boxes were sealed to avoid an exchange of gases with the environment.

## 3. Results

**Figure 1:**
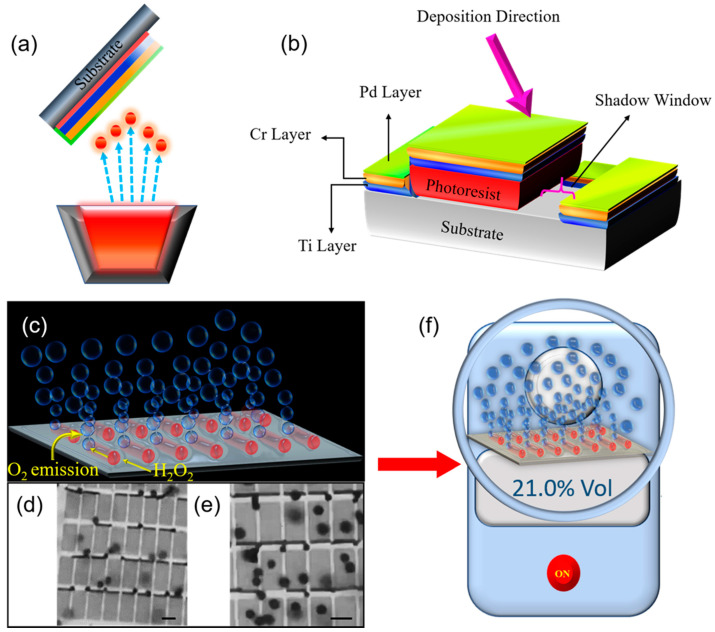
Oxygen generation using strain-engineered catalytic nanomembrane-based Ti/Cr/Pd microtubes; (**a**,**b**) Schematic representation of an angular e-beam deposition process; firstly the Ti layer (blue color), secondly the Cr layer (orange color) and thirdly the Pt layer (green color) are deposited on a sacrificial photoresist layer. (**c**) An illustration of microbubbles generation from tubular microreactor using decomposition of hydrogen peroxide into oxygen and water; (**d**,**e**) Optical microscopy images of rolled-up catalytic Ti/Cr/Pd microtube array integrated on Si substrate. The scale bars are 15 and 45 µm, respectively. The microtube’s oxygen bubbles generation in 8% v/v of hydrogen peroxide and 10% v/v Sodium dodecyl sulfate (SDS) surfactant; (**f**) Schematic image of oxygen concentration measurement by using an oxygen meter (in the air and in the closed volume of space containing an oxygen generator).

The field of nanomembranes is still in its infancy, which attracts research of novel materials properties, effects and functions. In this paper, the term “nanomembrane” is used to describe exclusively a freestanding film with nanoscale thickness below 100 nm and a large aspect ratio which can exceed 10,000. Our definition of the nanomembrane does not exclude the existence of nanopores, however, due to multiple rotations of rolled-up layers (and thick enough layers), we assume that fabricated microtubes do not contain nanopores. Oxygen evolution from the decomposition of hydrogen peroxide is studied using strain-engineered tubular catalytic Ti/Cr/Pd nanomembranes. Oxygen generation rate is recorded from the catalytic microtubes with different lengths in the range 15–100 µm using anionic surfactant and various H_2_O_2_ concentrations. An anionic surfactant sodium dodecyl sulfate (SDS), is the most common surfactant utilized in multiple cleaning and hygiene products. Schematic images of rolled-up nanotechnology on polymers fabrication method to produce strain-engineered Ti/Cr/Pd microtubes on the silicon substrate is shown in Figure 1a. When immersed in the hydrogen peroxide, fuel microtubes generate O_2_ microbubbles, while planar Pt in the same solution remains bubble-free. Optical microscopy images showing catalytic microtubes generating oxygen bubbles are displayed in Figure 1b,c, correspondingly. Multiple O_2_ bubbles emission from the uniform and align an array of catalytic microtube with lengths 15 and 45 µm length immersed in 8% v/v of hydrogen peroxide (H_2_O_2_) and 10% v/v SDS surfactant. The immersion of microtubes into the SDS surfactant and H_2_O_2_ solution generates the O_2_ bubbles during the H_2_O_2_ decomposition. The O_2_ bubbles grow and accumulate inside the microtubes. The ejected bubbles recoil and autonomously pump hydrogen peroxide fuel into microtubes. Figure 1f shows a schematic image of an oxygen meter for measurements of oxygen concentrations in the air and in the sealed boxes containing a catalytic microbubble generator.

**Figure 2:**
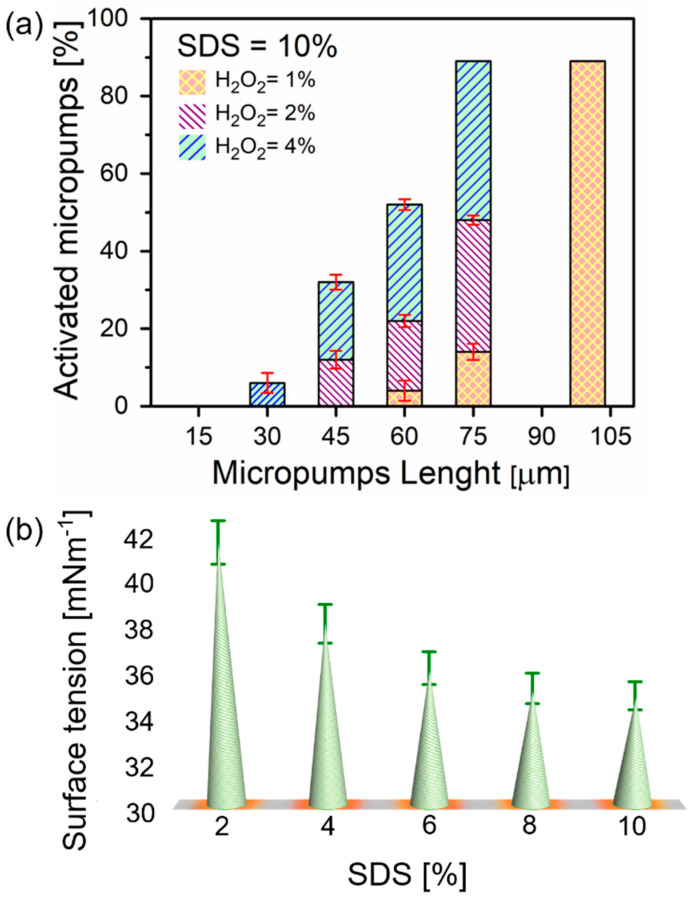
(**a**) Activated microtubes immersed in constant SDS (10% v/v) and in different concentrations of H_2_O_2_: 1%, 2% and 4% v/v, represented. The crossed right-tilted and left-tilted regions indicate different concentrations; (**b**) solutions surface tension containing different SDS concentrations (2–10% v/v).

Tube length, concentrations of surfactants, and hydrogen peroxide are of paramount importance for the oxygen bubbles generation [19]. Figure 2a illustrates microtubes with different lengths activated in various concentrations of peroxide fuel (activation here is the minimum peroxide concentration to start the generation of bubbles). The activation of microtubes immersed in 10% of the SDS surfactant solution is observed by the addition of H_2_O_2_: 1%, 2%, and 4%, respectively. It is demonstrated that in 1% concentration, microbubbles start to recoil for 60, 75, and 100 µm long microtubes. For 4% peroxide added to 60 µm long microtubes only 14% of 75 µm long microtubes became active. Moreover, 10% surfactant concentration influences the activation of 100 µm long microtubes, shown in the graph representing 89% activated microtubes. Hence, shorter 45 and 30 µm long tubes are activated in 2% and 4% concentrations of H_2_O_2_, respectively. The 12% activated fraction is noticed for 45 µm long and 6% for 30 µm long tubes. These results are similar to previously reported Ti/Cr/Pt microtubes [19]. It is noted that longer tubes, i.e., tubes with a high ratio of length to diameter, can confine better oxygen diffusion and lead to more efficient nucleation of bubbles [3]. Activation of a 15 µm microtube is required for a higher concentration of H_2_O_2_ due to its shorter length, i.e., using at least 6% of H_2_O_2_. From shorter tubes, molecular oxygen diffuses fast enough before the supersaturating of gaseous oxygen occurs—it is required for nucleation of bubbles.

The surface tension of hydrogen peroxide is one of the most important parameters that influence the generation of oxygen microbubbles [29]. Surfactants are known to stabilize microbubbles by accumulating at the air-liquid interface. Figure 2b shows surface tension measurements of hydrogen peroxide solution containing different concentrations of SDS performed using the maximum bubble pressure method. The maximum bubble pressure method can be used to measure the dynamic surface tension of solutions containing surfactants. Bubble pressure tensiometer pumps air through the capillary with the known diameter with known air pumping pressure. When the bubble diameter is equal to the diameter of the capillary, the bubble reaches maximum inside pressure. The surface tension is determined using Young-Laplace equation *σ* = Δ*P*·*R*_c_/2, where *σ* is the surface tension, Δ*P* is the maximum pressure drop and *R*_c_ is the radius of capillary. Addition of surfactants helps also achieve wetting of inner walls of microtubes by an aqueous hydrogen peroxide fuel, which otherwise can be blocked from flow into the tube. As expected, higher concentrations of SDS (2–10%) gradually decrease the surface tension of water. Particularly, it was reported that bubble generation ability depends on different surfactants, while microtubes are more active in solutions containing anionic than non-ionic and cationic surfactants [26]. How bubbles can nucleate, grow and recoil on the catalytic surface in the presence of different surfactants (e.g., anionic (sodium dodecyl sulfate, SDS), cationic (benzalkonium chloride, BACl) and non-ionic (Triton X)) and how this process can influence the speed of catalytic micromotors are hot topics of current investigations [27].

**Figure 3:**
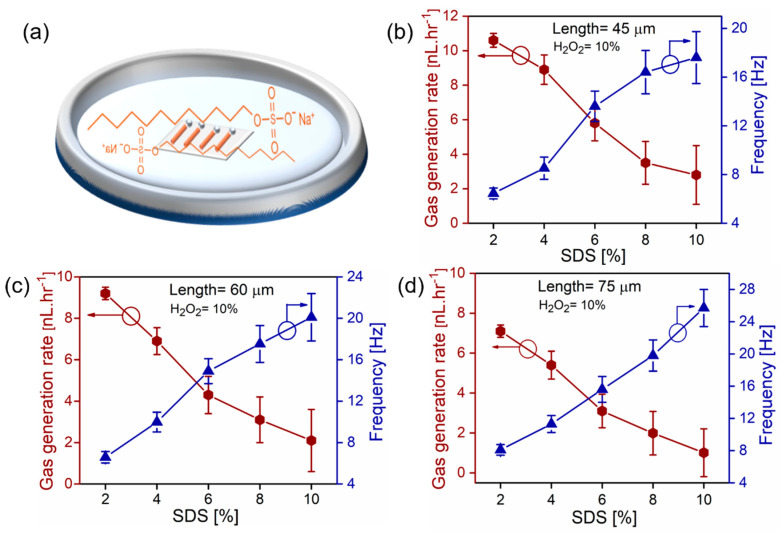
(**a**) Schematic image of SDS chemical formula and immersed catalytic microtubes in hydrogen peroxide fuel solution. (**b**–**d**) Dependence of oxygen bubbles frequencies and total gas generation rates (tube lengths 45, 60, and 75 µm) on concentrations of SDS surfactant (2–10% v/v). Hydrogen peroxide concentration is kept constant at 10% v/v.

Experiments of gas rate measurements are realized using different SDS concentrations and a constant 10% wt. H_2_O_2_, shown in Figure 3. It is revealed that higher concentrations of surfactants decrease the bubbles’ generation rate and total volumes of produced oxygen gas, which is in good agreement with previously reported platinum tubes [29]. Figure 3a shows a schematic image of microtubes in aqueous hydrogen peroxide solution with added surfactants. Figure 3b shows the total generated oxygen and frequencies of bubbles for 45 µm long tubes tested in 2–10 wt% v/v. SDS, 10% hydrogen peroxide. The amount of oxygen decreases almost linearly in the range 10.5–2.8 nL·hr^−1^, while bubble frequencies increase in the range 6.45–17.6 Hz. Figure 3c shows results for 60 µm long tubes, which produce 9.1–2.1 nL·hr^−1^ of oxygen with bubbles’ frequencies in the range 6.58–20.1 Hz. Similar trends are observed for 75 µm long tubes, shown in Figure 3d, where oxygen rates are in the range 7.1–1.01 nL·hr^−1^ with bubbles’ frequencies 8.1–25.7 Hz. These results reveal that shorter tubes in similar chemical conditions produce more total oxygen (despite their larger catalytic surface), while longer tubes generate oxygen bubbles at higher frequencies. Surfactants reduce surface tension and lead to higher frequencies of bubbles. However, the unexpectedly smaller total volume of oxygen is produced at higher concentrations of SDS concentrations. We attribute this observation to the nucleation and dynamic behavior of oxygen microbubbles in tubes. The previous studies about the influence of hydrogen peroxide fuel concentrations (with fixed tubular length and surfactant concentration) indicate that a transition peak exists [29]. Initially, higher concentrations of peroxide fuel lead to an increase of frequency of oxygen volume, however, at some critical point, the process reverses and less oxygen is generated. Figure 3 indicates that shorter tubes produce more oxygen gas at lower frequencies of bubbles. It is observed that bubbles are possibly connected by the neck to the tubular opening, which helps them grow to larger volumes and require less energy than bubbles split into smaller volumes inside the microtube. We hypothesize two possible explanation of this observation: (i) smaller recoiling microbubbles experience a larger total drag force during the migration/recoil through the microtube and (ii) the production of multiple smaller bubbles per time costs more energy than the generation of larger bubbles at more stable conditions, for example, bubble connection by the neck to the tubular opening. 

**Figure 4:**
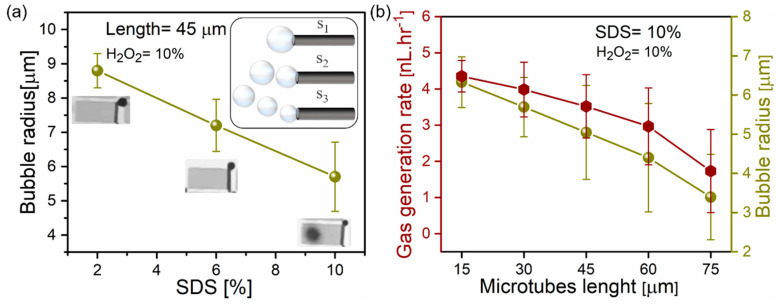
(**a**) Dependence of bubble radius and SDS surfactant concentration using constant tubular length (45 µm) and hydrogen peroxide concentration (10%). Insets are optical microscopy images indicating O_2_ bubbles detachment at different frequencies induced by the change of SDS concentrations 2, 6, 10% v/v, respectively. (**b**) Total oxygen generation and average bubble radius for 15, 30, 45, 60 and 75 µm long microtubes immersed in 10% SDS, 10% H_2_O_2_ solution.

Ti/Cr/Pd microtubes with a constant 45 µm length and 10% hydrogen peroxide are tested in different concentrations of SDS, shown in Figure 4a. The radius of ejected bubbles is reduced from an average value of 8.8 to 5.7 µm, indicating the reduction of solution surface tension. Inset images show optical micrographs and schematics of microtubes generating bubbles at different frequencies. Figure 4b represents how gas generation rates and average bubble radiuses change for tubes with different lengths: 15, 30, 45, 60 and 75 µm, correspondingly. Figure 4b demonstrates that despite larger catalytic area, longer tubes produce less total oxygen gas and the average bubble radius decreases. For the oxygen generation study in comparison of lengths, the shorter 15 and 30 µm generated 4.19 and 3.5 nL·hr^−1^ oxygen at 10% v/v fuel and 10%v/v SDS surfactant. Whereas 60 and 75 µm microtubes generated oxygen at lower rates 2.1 and 1.01 nL·hr^−1^ at 10% v/v fuel and 10% v/v SDS surfactant ejecting smaller bubbles. Shorter tubes produce larger bubbles with a higher oxygen gas generation rate. According to Young–Laplace equation bubble, internal pressure depends on the bubble size and the solution surface tension according to equation Δp = 2γ/r, where γ is the surface tension, r is the radius of bubble and Δp is the pressure difference across the fluid interface. Accepting the surface tension 41.5 × 10^−3^ N·m^−1^ (2%, SDS) and the bubble radius 8.8 µm, the Laplace pressure is 0.093 atm (negligible extra pressure). For a bubble with the radius 5.7 µm, the Laplace pressure is slightly higher 0.144 atm. On the other hand, if multiple bubbles nucleate in the tube, they migrate to a tubular opening and overcome additional drag force. If we neglect surface friction, the Stokes drag for the sphere is *F* = 6π*aμv*, where μ is the dynamic viscosity, a is the radius of the bubble and v is the bubble migration speed. It can explain higher oxygen rates produced by tubes, which are connected to growing bubbles by the gaseous neck for a longer time. Previously, we determined that shorter tubes are more efficient with bubble recoil, i.e., peroxide fuel pumping, than longer tubes. Shorter tubes also contain fewer bubbles, limited to tube volume and an aspect ratio. In contrast, longer tubes can generate a different number of bubbles because bubble nucleation sites can appear in a different position in the tube. For example, single or multiple bubbles can nucleate close to tubular opening or in the middle of the tube. We hypothesize that this factor leads to higher variability of both bubble frequencies and total oxygen produced volume. Figure 4 shows a statistical analysis of multiple tubes located on the individual sample. However, previously, in similar studies, large error bars were reported for longer catalytic microtubes [19,29]. 

**Figure 5:**
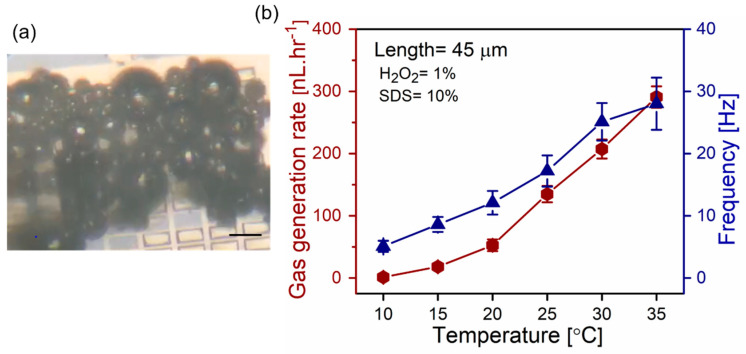
(**a**) Optical microscopy image of oxygen bubbles evolving from 45 µm catalytic tubes immersed in 1% v/v hydrogen peroxide fuel and 10% v/v SDS surfactant; (**b**) oxygen generation from catalytic microtube (left y-axis) and frequency of the microbubbles (right y-axis) at a constant SDS concentration (10% v/v) and H_2_O_2_ (1% v/v) versus temperature. Insets are optical micrographs of individual Ti/Cr/Pd tubes generating oxygen bubbles.

Higher temperature increases rates of hydrogen peroxide decomposition in the presence of a palladium catalyst. Figure 5 shows the temperature effect on the 45 µm long catalytic microtubes in 10% v/v surfactant and 1% v/v and H_2_O_2_ solution. Figure 5a is the optical micrograph of oxygen bubble generation at a temperature of 35 °C using 45 µm long tubes in 1% v/v peroxide and 10% v/v SDS. Subsequently, the temperature is set to 10, 15, 20, 25, 30, 35 °C to calculate the oxygen generation rates, as shown in Figure 5b. Less oxygen is generated at lower temperatures, i.e., 1.3 and 18.1 nL·hr^−1^ at 10 and 15 °C, correspondingly. The dramatic increase in oxygen generation is obtained from at higher temperatures, i.e., 20 and 35 °C, which is 52.6 to 290.91 nL·hr^−1^, correspondingly. Furthermore, bubble frequencies are increased from 5.1 to 28.1 Hz in 10–35 °C temperature range. Previously, the superfast motion of Ti/Cr/Pt microtubes was observed in similar conditions by increasing the temperature of the hydrogen peroxide solution [28]. These parameters enable the generation of oxygen at lower levels of hydrogen peroxide, i.e., more efficient bubble nucleation/generation, and achieve significantly higher oxygen rates by a relatively negligible change of solution temperature. Generally, temperature increases the rate of reaction due to a larger number of the colliding hydrogen peroxide molecules with the catalyst surface, which have required activation energy leading to more successful collisions for decomposition reaction to occur. Despite the reaction simplicity, many intermediate compounds can form and multiple elementary steps must be considered for better understanding of H_2_O_2_ decomposition over the Pd surface, for which we reserve our future study.

**Figure 6:**
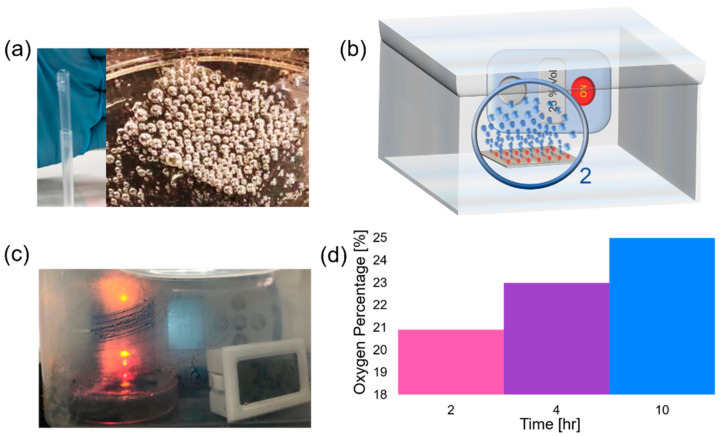
(**a**) Oxygen bubbles generation from silicon sample containing rolled-up Ti/Cr/Pd microtubes in 10% SDS and 1.5% v/v H_2_O_2_; (**b**) schematic image of oxygen bubble generator in a closed volume of space filled with air; (**c**) optical image of oxygen bubble generator prototype with humidified water condensed on the glass; (**d**) percentage of oxygen concentrations in the open air (pink region), large (purple region) and small (blue region) boxes with a hermetic seal.

We demonstrate the feasibility of oxygen accumulation in the closed volume of space towards the construction of a portable oxygen generator. Here, we measured oxygen concentration in ambient air equal to 20.9%. Subsequently, by placing our oxygen generator in large and small sealed boxes, higher oxygen concentrations were achieved. We assumed that some oxygen can be dissolved in water, but this oxygen amount remains low due to its limited solubility (at 20 °C approximately 0.9 mg of O_2_ can be dissolved in 100 mL of water). In our experiments, samples of a silicon substrate with integrated catalytic Ti/Cr/Pd microtubes immersed in solutions containing 10% v/v SDS and 1.5% H_2_O_2_ were used. Silicon substrate with integrated 45 µm long catalytic microtubes was immersed into 10% v/v SDS and 1.5% H_2_O_2_ at 22 °C. The optical image of the oxygen generator in the transparent container is shown in Figure 6c. Initially, 20.9% oxygen is observed that represents an ambient air oxygen concentration (Figure 6d, pink color region). After placement of the sample in the box (l × h × w= 16 × 10 × 10 cm) oxygen concentration increased to 23% in four hours (purple color region, Figure 6d). After placement of the sample in the box (l × w × h = 8 × 6 × 6 cm) 25% oxygen was measured in four hours (blue color region, Figure 6d). In both cases, before samples were placed in boxes, the hydrogen peroxide solution was heated to 35 °C. Subsequently, samples were sealed in boxes and left in ambient conditions for four hours to measure oxygen concentrations. This is the proof-of-concept result indicating that it is feasible to increase the concentration of oxygen in local space in time using our catalytic oxygen generator. Moreover, the Pd catalyst does not degrade during several hours of testing.

## 4. Conclusions

In summary, the controlled oxygen evolution and bubbles frequencies are studied using Ti/Ni/Pd microtubes immersed in different SDS surfactant and hydrogen peroxide concentrations. Chemical oxygen generators can be designed and well understood on the level of individual tubular microreactors immersed in the fuel solution with required chemical compositions. Firstly, geometrical parameters of tubular microreactors that influence reaction-diffusion processes have to be considered. Longer microtubes with a high ratio of length to diameter can confine diffusion of molecular oxygen during the decomposition of hydrogen peroxide. It leads to a supersaturation of gaseous oxygen molecules supersaturation and nucleation of microbubbles in the tubular microcavity. Molecular diffusion of shorter microtubes is significantly shorter, which leads to rapid diffusion of molecular oxygen from microtubes and thus, shorter microtubes require higher rates of hydrogen peroxide decomposition to achieve the threshold level of oxygen supersaturation. This study shows that higher SDS surfactant concentrations lead to lower surface tension, lower bubble radius, and higher frequencies. However, smaller bubbles produce less total oxygen gas. Previously, it was determined that at fixed surfactant concentration and tubular length, oxygen generation peak exists in different solutions of hydrogen peroxide [29]. Initially, an increase of hydrogen peroxide concentration produces higher frequencies of bubble recoil and total produced oxygen gas. Then, however, bubbles are decreased in size and less total volume of oxygen is generated. We attribute this observation to friction force experienced by migrating/recoiling bubbles and energy favorable conditions (initially bubbles are connected by the gaseous neck, then bubbles are split into multiple nucleation points within the tube). More quantitative investigations are required to better understand this observation. Another important point is the threshold concentration of hydrogen peroxide required for bubbles generation: shorter tubes require a significantly higher concentration of hydrogen peroxide to generate bubbles than longer tubes. Although one sample cannot provide enough oxygen for portable oxygen generators, understanding of oxygen release on the level of individual tubular microreactors can be used to develop scalable oxygen generators in the future. The temperature has a pronounced effect on the rate of the catalytic reaction of hydrogen peroxide decomposition: 45 µm microtubes immersed in 10% v/v SDS and 1% H_2_O_2_ at temperature 35 °C, produce 290.9 nL·hr^−1^ of oxygen. While pressurized oxygen in tank form and oxygen concentrators are ubiquitous in healthcare (e.g., therapy against COVID-19), however not every hospital, especially in developing countries, can afford such expensive and complex oxygen delivery platforms. Recently, novel cheaper, cleaner and decentralized methods to produce hydrogen peroxide have been demonstrated, such as recently developed electrochemical reactors that require only oxidized carbon nanoparticle-based catalyst, air, water and electricity (solar panel powered) to synthesize H_2_O_2_ with high Faradaic efficiencies [43]. Portable catalytic oxygen generators are of high advantage for a controllable release of oxygen on demand including biomedical, chemical and emergency applications.
